# Role of Microvesicles in the Spread of Herpes Simplex Virus 1 in Oligodendrocytic Cells

**DOI:** 10.1128/JVI.00088-18

**Published:** 2018-04-27

**Authors:** Raquel Bello-Morales, Beatriz Praena, Carmen de la Nuez, María Teresa Rejas, Milagros Guerra, Marcos Galán-Ganga, Manuel Izquierdo, Víctor Calvo, Claude Krummenacher, José Antonio López-Guerrero

**Affiliations:** a Universidad Autónoma de Madrid, Departamento de Biología Molecular, Cantoblanco, Madrid, Spain; b Centro de Biología Molecular Severo Ochoa, CSIC-UAM, Cantoblanco, Madrid, Spain; c Universidad Autónoma de Madrid, Facultad de Medicina, Madrid, Spain; d Department of Biological Sciences and Department of Molecular and Cellular Biosciences, Rowan University, Glassboro, New Jersey, USA; Northwestern University

**Keywords:** extracellular vesicles, microvesicles, oligodendrocytes, viral spread, herpes simplex virus

## Abstract

Herpes simplex virus 1 (HSV-1) is a neurotropic pathogen that can infect many types of cells and establishes latent infections in the neurons of sensory ganglia. In some cases, the virus spreads into the central nervous system, causing encephalitis or meningitis. Cells infected with several different types of viruses may secrete microvesicles (MVs) containing viral proteins and RNAs. In some instances, extracellular microvesicles harboring infectious virus have been found. Here we describe the features of shedding microvesicles released by the human oligodendroglial HOG cell line infected with HSV-1 and their participation in the viral cycle. Using transmission electron microscopy, we detected for the first time microvesicles containing HSV-1 virions. Interestingly, the Chinese hamster ovary (CHO) cell line, which is resistant to infection by free HSV-1 virions, was susceptible to HSV-1 infection after being exposed to virus-containing microvesicles. Therefore, our results indicate for the first time that MVs released by infected cells contain virions, are endocytosed by naive cells, and lead to a productive infection. Furthermore, infection of CHO cells was not completely neutralized when virus-containing microvesicles were preincubated with neutralizing anti-HSV-1 antibodies. The lack of complete neutralization and the ability of MVs to infect nectin-1/HVEM-negative CHO-K1 cells suggest a novel way for HSV-1 to spread to and enter target cells. Taken together, our results suggest that HSV-1 could spread through microvesicles to expand its tropism and that microvesicles could shield the virus from neutralizing antibodies as a possible mechanism to escape the host immune response.

**IMPORTANCE** Herpes simplex virus 1 (HSV-1) is a neurotropic pathogen that can infect many types of cells and establishes latent infections in neurons. Extracellular vesicles are a heterogeneous group of membrane vesicles secreted by most cell types. Microvesicles, which are extracellular vesicles which derive from the shedding of the plasma membrane, isolated from the supernatant of HSV-1-infected HOG cells were analyzed to find out whether they were involved in the viral cycle. The importance of our investigation lies in the detection, for the first time, of microvesicles containing HSV-1 virions. In addition, virus-containing microvesicles were endocytosed into CHO-K1 cells and were able to actively infect these otherwise nonpermissive cells. Finally, the infection of CHO cells with these virus-containing microvesicles was not completely neutralized by anti-HSV-1 antibodies, suggesting that these extracellular vesicles might shield the virus from neutralizing antibodies as a possible mechanism of immune evasion.

## INTRODUCTION

Herpes simplex virus 1 (HSV-1) is a highly prevalent (1) human pathogen belonging to the neurotropic alphaherpesviruses that can establish latency in neurons (2). After primary infection of epithelial cells, this virus spreads to neurons and establishes latent infections in the trigeminal ganglia. Under certain circumstances, HSV-1 may cause severe pathologies, such as keratoconjunctivitis or encephalitis (3). HSV-1 is also an increasing cause of genital herpes (4, 5). HSV-1 has the ability to enter many different hosts and cell types (6) using different receptors and different pathways: plasma membrane fusion at neutral pH and low-pH-dependent or low-pH-independent endocytosis (reviewed in references 7 to 10). Regardless of the pathway, HSV glycoproteins, such as the receptor-binding glycoprotein D (gD), the fusion modulator complex gH/gL, and the fusion effector gB, are essential for virion entry. Antibodies (Ab) raised against these glycoproteins may have strong neutralizing activities (11–13).

Regarding maturation and egress, four major stages have been proposed to describe these processes: capsid assembly and DNA packaging in the nucleus; primary envelopment and deenvelopment at the nuclear envelope; tegumentation and secondary envelopment in the cytoplasm; and, finally, exocytosis of viral particles at the plasma membrane and/or cell-to-cell transmission at cell junctions (14). A major mode of HSV-1 transmission in human tissues is cell-to-cell spread, that is, the direct passage of progeny virus from an infected cell to an adjacent one (15). It is widely accepted that this mechanism of spread represents an immune evasion strategy, since it protects the virus from immune surveillance (15). However, as mentioned above, HSV-1 may use several modes of spread to pass from infected to uninfected cells (7). Many aspects concerning the process of viral spread, for instance, the mechanisms of viral egress from epithelial cells and spread to neurons and vice versa (16), are not completely understood yet. Clarifying the mechanisms of viral dissemination and subsequent entry into neighboring cells remains a necessary step to understand the viral cycle in the host (7).

In this context, secreted vesicles have emerged as a new object of attention because of their ability to participate in the intercellular communication process during viral infections. Extracellular vesicles (EVs) are a highly heterogeneous group of secreted membrane vesicles which have been isolated from most cell types and biological fluids (17–20). Three major subgroups of EVs have been identified: apoptotic bodies; microvesicles (MVs), which derive from the shedding of the plasma membrane; and exosomes, which are intraluminal vesicles released to the extracellular space upon fusion of multivesicular bodies (MVBs) with the plasma membrane (19, 21). Exosome are typically 30 to 100 nm in diameter, while MVs have a heterogeneous size ranging from 100 nm to 1 μm in diameter (18, 22). EVs have been shown to be involved in numerous physiological and pathological processes, such as inflammation and the immune response, cell adhesion, coagulation, waste management, tumor progression, and viral spread (19, 20, 23–27).

Oligodendrocytes (OLs) are the myelin-forming cells of the central nervous system (CNS) (28–31). The myelin sheath is an electrically insulating layer that surrounds axons in both the central and peripheral nervous systems, allowing saltatory conduction of action potential. All neural cell types secrete EVs (32), which have a central role in processes such as myelinization (33) or regulation of synaptic activity (22, 34, 35) and might be involved in the pathogenesis of several neurodegenerative diseases (32, 36, 37) or, on the contrary, in neuroprotection (22). Indeed, OLs secrete exosomes carrying myelin proteins, such as proteolipid protein (PLP), the major myelin protein in the CNS; 2′3′-cyclic nucleotide phosphodiesterase (CNPase); myelin basic protein (MBP); and myelin oligodendrocyte glycoprotein (MOG), as well as a number of enzymes, such as glycolytic or oxidative stress-alleviating enzymes (38, 39). Previous works carried out by our group have shown that OLs are highly susceptible to HSV-1 infection (40). Subsequent works revealed that oligodendrocyte precursor cells (OPCs) and cells of the human oligodendroglial HOG cell line cultured under differentiation conditions become more susceptible to HSV-1 (41), a fact that was consistent with the later finding that PLP, a myelin protein upregulated throughout differentiation, is involved in viral entry (42). These findings support the hypothesis that OLs might be a more susceptible target of HSV-1 infection in glial cells undergoing differentiation, a fact that could be relevant in HSV-1 pathogenicity.

EVs derived from OLs have also been involved in such processes, and there is increasing evidence for the role of EVs in myelination and neuron-glia communication (33, 35, 43). EVs have also been involved in viral infection (44–47) as a means for viruses to enter host cells, enhance spread, or evade the host immune response (48, 49). Two major functions of EVs during viral infections are the transfer of viral genomes into target cells and the reinforcing of infection by changing the physiology of target cells (49). Production of secreted vesicles by HSV-1-infected cells has been known for a long time. The first to be discovered, named L-particles, are similar to the virions in appearance, but they lack the viral nucleocapsid and the genome and therefore are not infectious (50). However, L-particles have been shown to facilitate HSV-1 infection by delivering viral proteins and the cellular factors needed for virus replication and immune evasion (51). Thus, it has been argued that the similar functions observed for *vhs* and α-TIF between virions and L-particles suggest that viral attachment, fusion, and release of tegument proteins are the same for both (52). In addition, L-particles share similar assembly and egress pathways with virions, suggesting that the tegument and glycoproteins are sufficient to prompt secondary envelopment (14). It has been demonstrated that functional viral proteins can be transferred to uninfected bystander cells via L-particles, a process that may indicate a strategy for viral immune escape (53). Other particles, the previral DNA replication-enveloped particles (PREPs) (54), are morphologically similar to L-particles, but they differ in their relative protein compositions.

However, to date, there is no evidence of HSV-1 virions being packaged inside EVs (51). Here, we propose a novel role for MVs in HSV-1 spread. Our findings indicate for the first time that HSV-1 virions may be transferred from infected to uninfected cells via MVs. By means of transmission electron microscopy (TEM), we detected microvesicles containing HSV-1 virions. In addition, we found that the nonpermissive Chinese hamster ovary (CHO) cell line was susceptible to HSV-1 infection only after inoculation with virus-containing MVs previously isolated from a supernatant of infected HOG cells. Moreover, unlike infection of cells of the oligodendrocytic HOG cell line, infection of CHO cells was not neutralized when virus-containing MVs were inoculated after being incubated with anti-HSV-1 antibodies; that is, an anti-HSV-1 polyclonal antibody which completely neutralized the entry of free virions into HOG cells failed to efficiently block infection of CHO cells by virus-containing MVs. Taken together, these results suggest that MVs secreted by HOG cells infected with HSV-1 might be involved in viral spread and may contribute to avoiding immune surveillance.

## RESULTS

### Characterization of MVs from cell culture supernatants of HOG cells.

**FIG 1:**
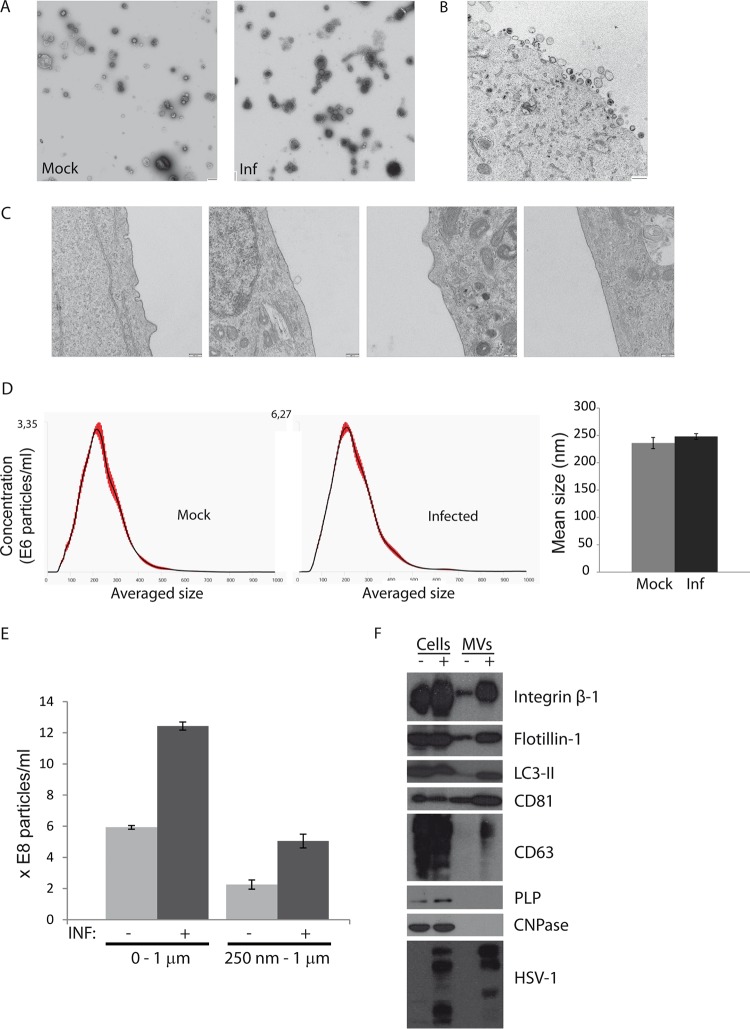
Isolation of MVs from the cell culture supernatants of HOG cells. (A) MVs were isolated by differential centrifugation from the supernatants of HOG cells that had been mock infected (Mock) and infected (Inf) with HSV-1 at an MOI of 1 for 24 h. MVs were adsorbed onto collodion-carbon-coated copper grids and negatively stained with aqueous uranyl acetate. Bars = 500 nm. (B) HOG cells were incubated at 4°C for 1 h with MVs isolated from HSV-1-infected HOG cells and then for 15 min at 37°C. After that the cells were fixed and processed for electron microscopy. The image shows the presence of virions and a heterogeneous group of MVs attached to the plasma membrane. (C) Four random images corresponding to the MV-untreated cell control, showing the plasma membrane free of attached vesicles. (D) The pellet containing the MVs obtained as described above was resuspended and kept at 4°C in serum-free DM before its analysis by nanoparticle tracking analysis (NTA). The plots and histogram show the average size of MVs. (E) Histograms representing the concentration of particles (×10E8) per milliliter show an increase in the concentrations of particles obtained from infected cells (dark bars) compared to mock-infected ones (light bars). To exclude the possibility of the presence of isolated virions, we also performed the analysis with particles with a size larger than 250 nm. (F) Immunoblots show the presence of integrin β-1, flotillin-1, LC3-II, and CD81 in MVs isolated from mock-infected (−) and infected (+) cells. CD63 was observed in the MV fraction isolated from infected cells. The myelin proteins CNPase and PLP were not detected in MVs. The bands corresponding to the anti-HSV-1 antibody are also shown.

To isolate MVs, HOG cells infected and mock infected with HSV-1 at a multiplicity of infection (MOI) of 1 were cultured with differentiation medium (DM) (41). The lack of serum in DM prevents contamination of our MV preparation with EVs originating from fetal bovine serum (FBS) (55). After 24 h of infection, 30 ml of supernatant was collected. MVs were isolated by differential centrifugation following a series of centrifugation steps at 4°C: first at 400 × *g* for 10 min, then at 2,500 × *g* for 15 min, and, finally, at 10,000 × *g* for 30 min. MVs isolated from infected and mock-infected cells were processed for electron microscopy using a methylcellulose-uranyl acetate mixture for staining and embedding (56). We observed heterogeneous MVs ranging from approximately 100 nm to 1 μm from both infected and mock-infected HOG cells (Fig. 1A), and numerous virions were present in the MV fraction obtained from infected cells.

Next, MVs from infected cells were layered onto HOG cells at 4°C for 1 h and incubated for 15 min at 37°C before fixation and processing for electron microscopy by conventional Epon embedding methods. Figure 1B shows the presence of virions and a heterogeneous group of MVs attached to the plasma membrane. The MV-untreated cell control showed that the plasma membrane was free of attached vesicles or, exceptionally, that vesicles were being shed from the membrane (Fig. 1C).

To quantify the concentration and size of MVs, we performed nanoparticle tracking analysis (NTA). Results showed a similar average size of MVs isolated from infected and mock-infected cells, which was found to be about 250 nm (Fig. 1D). Nevertheless, the concentration of particles from infected cultures was higher than that from mock-infected ones (Fig. 1E). To exclude the possibility of the presence of isolated virions, we also performed an assessment of particles with a size larger than 250 nm. We observed an increase in total vesicles as well as in large vesicles (MVs without free virions) in samples from infected HOG cells.

Immunoblot assay showed the presence of integrin β-1 and flotillin-1 in MVs obtained from both infected and mock-infected cells, whereas the myelin proteins CNPase and PLP were absent in this fraction. The MVs were also positive for the exosomal markers CD63 and CD81, as well as the autophagy marker LC3-II (Fig. 1F). The increment in signals corresponding to MVs isolated from infected cells might be due, at least in part, to the increment in MV production in these cells compared to the mock-infected ones.

**FIG 2:**
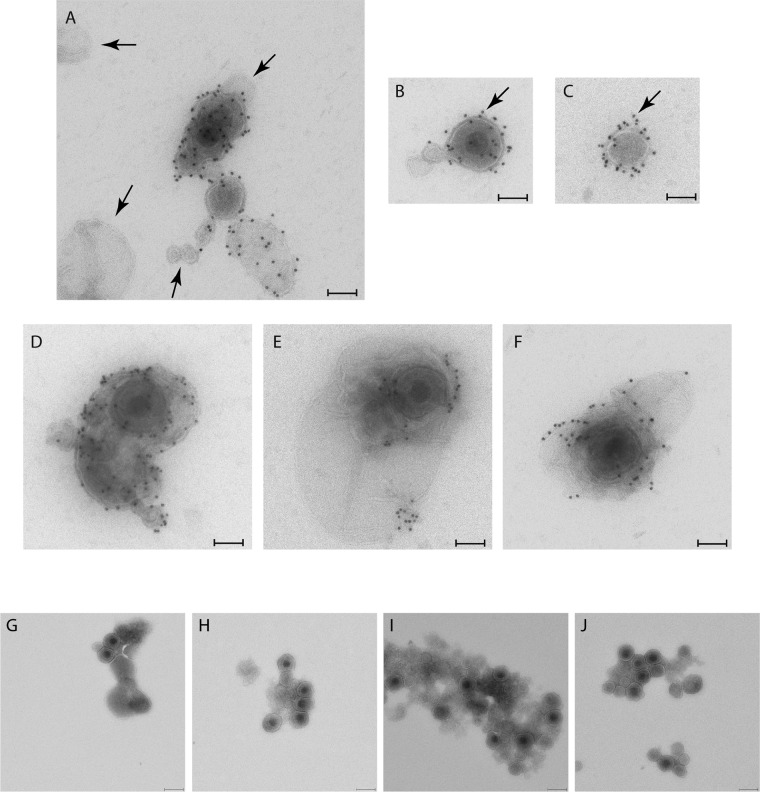
Immunoelectron microscopy of MVs obtained from HOG cells infected with HSV-1. MVs from HOG cells infected with HSV-1 were fixed and processed for immunoelectron microscopy. (A) Vesicles labeled with an anti-HSV-1 antibody coupled to colloidal gold can be seen, although not all vesicles (arrows) are labeled with viral proteins. (B and C) In isolated virions (B) and MVs (C), viral proteins exposed outside the membrane (arrows) can be detected. (D to F) Images show the presence of MVs containing virions. (G to J) Four random images corresponding to the control of centrifugation, in which a mixture of virions with MVs was centrifuged to exclude the possibility that the presence of virions inside MVs was artifactual. Although the images corresponding to the centrifugation of virions with vesicles showed a compact group of vesicles and viral particles tightly situated, MVs containing virions were not detected. Bars = 90 nm (A to F) and 200 nm (G to J).

To determine whether MVs isolated from HOG cells infected with HSV-1 contained viral proteins and/or virions, MVs were isolated and processed for immunoelectron microscopy with an antibody against HSV-1. Numerous vesicles were labeled positive with a rabbit anti-HSV-1 antibody followed by protein A coupled to 10-nm colloidal gold, although not all vesicles were labeled (Fig. 2A). In isolated virions and MVs (Fig. 2B and C), viral proteins exposed on the membrane were observed, and MVs containing virions (Fig. 2D to F) were also labeled. To assess the specificity of the anti-HSV-1 antibody, MVs from mock-infected cells were also tested and, as expected, showed no labeling.

The relative centrifugal force required to isolate MVs is frequently between 10,000 and 20,000 × *g* (57–59), and centrifugation at 10,000 × *g* for 30 min, the method used in our study, is a widely used protocol (56, 60–63). However, to control the effect of centrifugation, we have included throughout our experiments controls to avoid inaccurate results. In this step, we included a control in which we centrifuged a mixture of virions with MVs under the same conditions of medium, volume, temperature, and time and the same parameters of centrifugation to exclude the possibility that the presence of virions inside MVs was artifactual. Although centrifugation of virions with vesicles yielded a compact group of vesicles and viral particles tightly situated, MVs containing virions were not detected in these controls (Fig. 2G to J).

### Infection by MVs purified from HSV-1-infected HOG cells.

**FIG 3:**
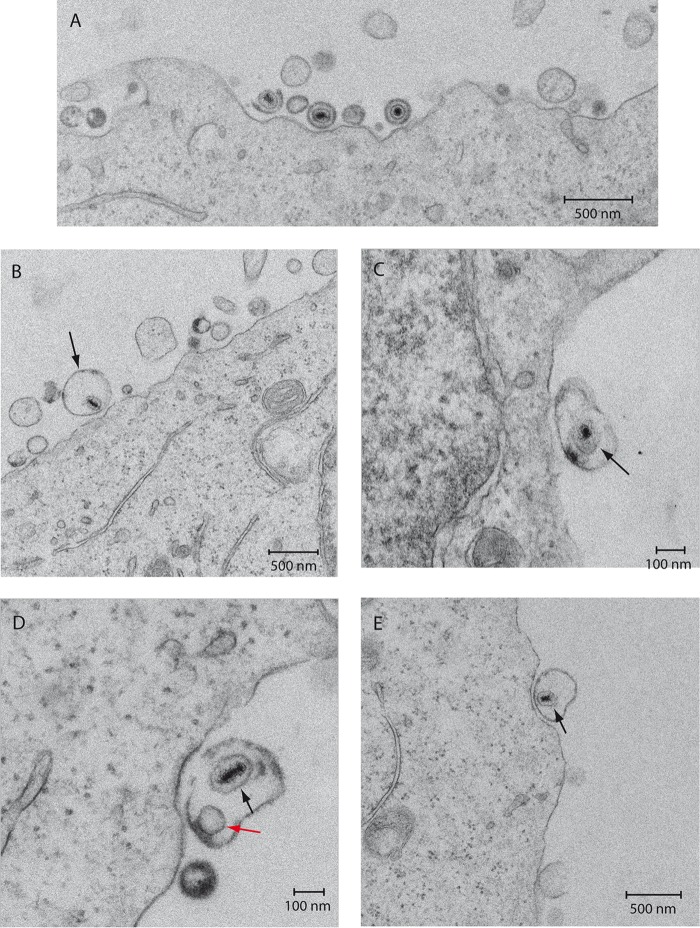
Electron microscopy of HOG cells incubated with MVs. MVs from HOG cells infected with HSV-1, isolated as described in the text, were layered onto HOG cells and incubated at 4°C for 1 h. After that, they were incubated for 15 min at 37°C, fixed, and processed for electron microscopy. (A and B) Although the majority of MVs lacked virions (A), occasionally, virion-containing MVs were observed (B). (C to E) Most of the virion-containing MVs enclosed only one viral particle, which lacked, in most cases, an organized envelope (black arrows). Occasionally, more virions or other vesicles (red arrow) were also found in the virion-containing MVs (D). MVs with inner virions attached to incipient cell membrane invaginations, resembling the initiation of an endocytic process, were also observed (D and E).

MVs isolated from HOG cells infected with HSV-1 were layered onto HOG cells and incubated at 4°C for 1 h, followed by incubation at 37°C for 15 min. For quantitation of virion-containing MVs, 5 representative fields were imaged with a TEM and MVs were visually counted. Although the majority of MVs seemed to lack virions (Fig. 3A), virion-containing MVs were occasionally observed (Fig. 3B). The quantitative analysis of these vesicles showed that 5.17% ± 0.40% of MVs contained virions. Most of the virion-containing MVs enclosed only one viral particle, which in most cases appeared to lack an envelope (Fig. 3B to E). Occasionally, more than one virion or other vesicles were also found in MVs (Fig. 3D). MVs with inner virions attached to incipient cell membrane invaginations were also observed (Fig. 3D and E), resembling the initiation of an endocytic process.

**FIG 4:**
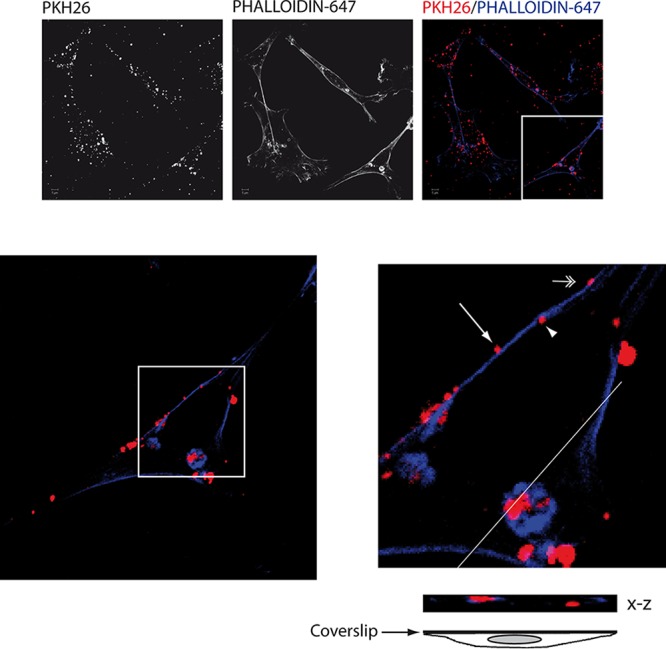
Immunofluorescence analysis of HOG cells incubated with MVs. MVs from HSV-1-infected HOG cells were stained with the red dye PKH26 and added to naive HOG cells. After 2 h of incubation at 37°C, the cells were fixed and stained with Alexa Fluor 647-phalloidin to visualize the contour of the cells. Images showed MVs colocalizing with the plasma membrane (double-headed arrow). In addition, MVs attached to the plasma membrane (arrow) and inside the cells (arrowhead) can be observed in the magnified region corresponding to the white square, suggesting a process of endocytosis of MVs. Images correspond to a 6-μm confocal plane.

To assess whether MVs were susceptible to be endocytosed, we isolated MVs from noninfected HOG cells, and then they were stained with the red dye PKH26 and added to naive HOG cells. After 2 h of incubation at 37°C, cells were fixed and stained with Alexa Fluor 647-phalloidin to visualize the contour of the cells by confocal fluorescence microscopy. As seen in Fig. 4, numerous MVs (in red) were observed to be attached to HOG cells (in blue), and MVs colocalizing with the plasma membrane were also observed (in magenta). In addition, through analyzing 6-μm confocal planes, we detected MVs inside the cells, suggesting a process of endocytosis of MVs (Fig. 4).

**FIG 5:**
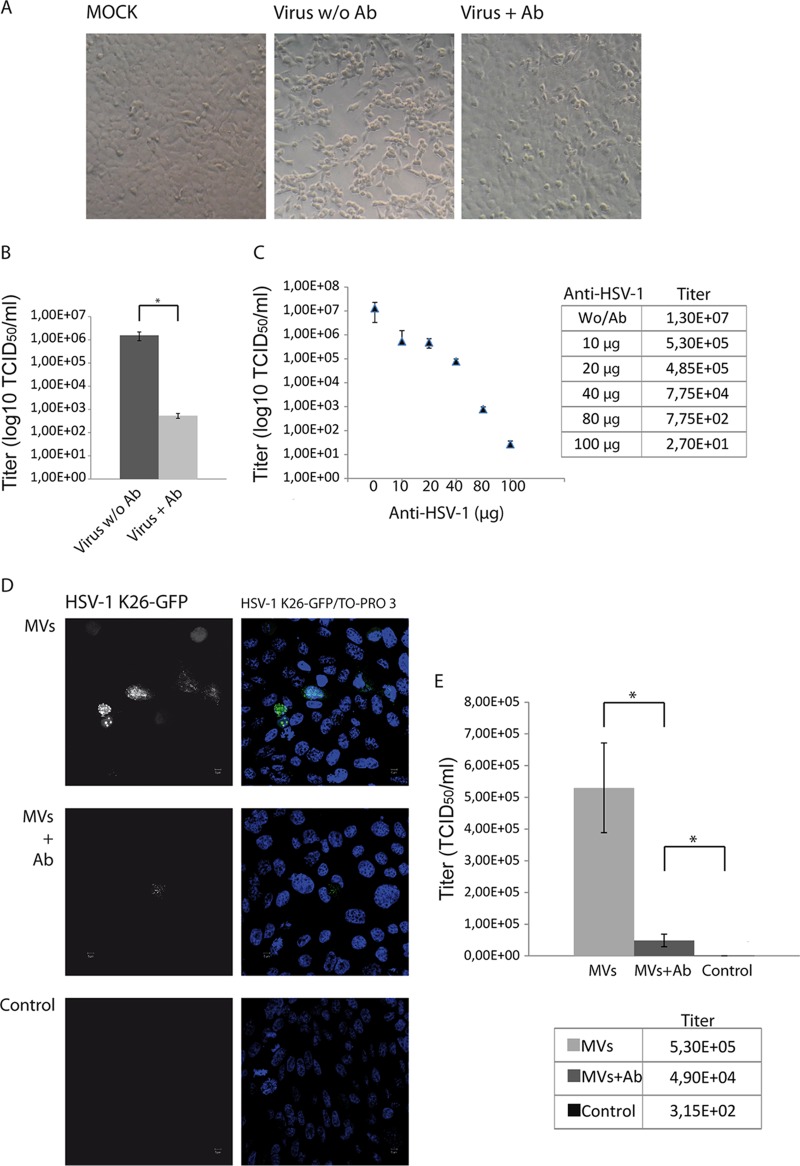
Infection of nonpermissive CHO-K1 cells with MVs obtained from HOG cells infected with HSV-1 K26-GFP. To evaluate the neutralization capacity of the anti-HSV-1 antibody, HOG cells were infected with K26-GFP incubated with or without an anti-HSV-1 polyclonal antibody. (A and B) The figure shows the lack of a cytopathic effect (A) and a significant decrease in viral production (B) in cells infected with antibody-incubated virus. (C) To quantify the neutralization capacity of the anti-HSV-1 antibody, we performed a titration assay as described in Materials and Methods. The figure shows that 100 μg of antibody can neutralize 1 × 10E8 TCID_50_. HOG cells were infected with K26-GFP. MVs from these HOG-infected cells, obtained as described above, were incubated with or without anti-HSV-1 antibody and subsequently added to receptor-deficient CHO-K1 cells, regarded as nonpermissive for HSV-1 infection, and incubated again for 1 h at 37°C. After adsorption, the cells were left for 24 h with DM. (D) Immunofluorescence images show the presence of infected CHO cells, suggesting that MVs from HOG-infected cells had transferred infectious virus to the nonpermissive CHO-K1 cell line. Infection of cells with antibody-incubated virus (MVs + Ab) showed a marked decrease, but despite that, infected CHO cells could be observed. A control for CHO cells infected directly with K26-GFP did not show a significant number of infected cells (Control). (E) Titration of viral production of CHO cells infected as described in the legend to panel D showed the same phenomenon: the susceptibility of CHO cells to MV-mediated infection (MVs), the existence of a significant viral production even in the presence of a neutralizing antibody (MVs + Ab), and the lack of significant viral production in CHO cells infected with the virus (Control). *, *P* < 0.05.

Given that MVs might be endocytosed, we then wondered whether these MVs could spread infection to naive cells. To verify this point, we took advantage of CHO cells, which are highly resistant to HSV-1 entry infection due to the lack of gD receptors (64, 65). Consequently, the use of CHO-K1 cells excludes the possibility of infection with virions but may allow MV-mediated infection, since the endocytosis of MVs may not require viral receptors. Here, HOG cells were infected with HSV-1 strain K26 fused with green fluorescent protein (GFP) (K26-GFP), and MVs were isolated as described earlier. CHO-K1 cells were inoculated with these MVs and incubated for 24 h to allow GFP expression. Immunofluorescence images showed the presence of infected GFP-positive CHO cells (Fig. 5D), indicating that MVs from HOG-infected cells had transferred infectious virus to nonpermissive CHO-K1 cells. Titration of viral production of CHO cells infected with these MVs confirmed the susceptibility of CHO cells to MV-mediated infection (Fig. 5E). As expected, CHO cells infected directly with K26-GFP virions did not show a significant number of infected cells (control). As we have stated earlier, the MV preparations were not free of virions. Therefore, in order to design appropriate infection controls, we had to quantify the number of virions that were present in the MV fraction. To design this control, we calculated the difference between the viral titer before and the viral titer after the 10,000 × *g* centrifugation. Thus, we could calculate the number of infectious virions that had been pelleted along with the MVs. The infectious titer of MV preparations obtained from HOG cells was 1.1 × 10E7 50% tissue culture infective doses (TCID_50_)/ml, as determined on Vero cells. We used the same amount of infectious HSV-1 K26-GFP virions in our control (Fig. 5D, bottom) to match the MV inoculation conditions. Thus, the absence of infection in CHO cells exposed to an amount of free virions comparable to what is found in MV preparations (Fig. 5D bottom) suggests that the contribution of free visions in infection of CHO cells exposed to MVs is negligible.

We then tested whether MVs isolated from HSV-1-infected HOG cells were susceptible to be detected and blocked by neutralizing antibodies directed against HSV-1. Neutralizing antibodies prevent attachment of virions to cells, block necessary receptor interactions after attachment, or interfere with any other necessary step in the entry of viruses, such as coreceptor engagement or endocytosis (66). In this assay, we used a rabbit polyclonal anti-HSV-1 antibody as described in Materials and Methods. This polyclonal antibody was produced in rabbit with strain F of HSV-1 as an immunogen and presents cross-reactivity with HSV-2. To determine the neutralizing potential of this polyclonal antibody on free virions, we preincubated HSV-1 K26-GFP (1 × 10E8 TCID_50_) with 80 μg of anti-HSV-1 antibody for 1 h at room temperature in a final volume of 200 μl of serum-free Dulbecco modified Eagle medium (DMEM). After 1 h of preincubation, the mixture was layered on HOG cells seeded on coverslips for immunofluorescence and incubated at 37°C for 1 h. After adsorption, the inoculum was removed and the cells were incubated at 37°C for 24 h. A control without antibody was included. The lack of a cytopathic effect (Fig. 5A) and a significant decrease in viral production (Fig. 5B) in cells infected with antibody-incubated virus confirmed the robust neutralizing activity of this antibody. To further quantify the neutralization capacity of the anti-HSV-1 antibody, we performed a titration assay as described in Materials and Methods, finding out that 100 μg of antibody reduced the titer from 1.3 × 10E7 to 2.7 × 10E1 (about 5 × 10E5-fold) (Fig. 5C). Once we determined the amount of antibody necessary to neutralize free virus, we carried out the neutralization assay on MVs. To do that, HOG cells were infected with K26-GFP. MVs from these HOG-infected cells, obtained as described above, were incubated with or without anti-HSV-1 antibody using the conditions determined above. MVs mixed with antibodies were added to CHO-K1 cells and incubated again for 1 h at 37°C. After adsorption, the inoculum was removed and the cells were washed and further incubated for 24 h in DM to allow for GFP expression. Immunofluorescence images showed that infection of cells with antibody-incubated virus decreased significantly (Fig. 5D, MVs + Ab). Nevertheless, despite the presence of an excess of neutralizing antibody, infected CHO cells could readily be observed. The analysis of viral production showed that 100 μg of antibody reduced the titer from 5.3 × 10E5 to 4.9 × 10E4 (about 10-fold) (Fig. 5E). Note that the same amount of antibody reduced free virus infection by 5 × 10E5-fold (Fig. 5C). Therefore, there was a significant fraction of MV-mediated infectivity that could not be neutralized even with a highly effective dose of antibody. In conclusion, these data indicate that MVs are not neutralized as effectively as virions. Finally, a control of CHO cells infected directly with K26-GFP did not show a significant number of infected cells (Fig. 5D, bottom). In summary, titration of viral production of CHO cells exposed to MVs or free virus (Fig. 5E) confirmed the susceptibility of receptor-negative CHO cells to MV-mediated infection but not to free virus infection and suggests that MVs can escape neutralization to spread infection.

### Analysis of MVs from nonoligodendrocytic cell cultures infected with HSV-1.

**FIG 6:**
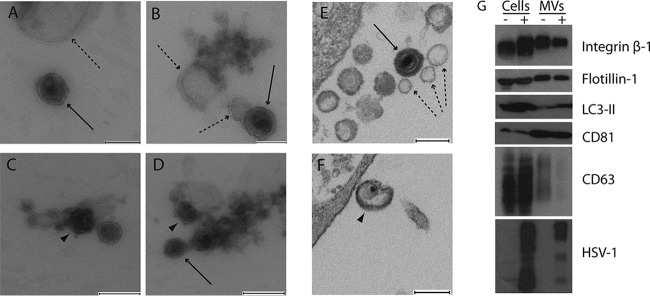
Analysis of MVs secreted by HeLa cells. MVs from HeLa cells infected with HSV-1 at an MOI of 1 for 24 h were isolated by differential centrifugation from the supernatants of HeLa cell cultures as previously described. (A to D) MVs were adsorbed onto collodion-carbon coated-copper grids and negatively stained and embedded with methylcellulose-uranyl acetate as described in Materials and Methods. (E and F) HeLa cells were incubated at 4°C for 1 h with MVs isolated from HSV-1-infected HeLa cell cultures and then for 15 min at 37°C. After that, the cells were fixed and processed for electron microscopy. Images show the presence of virions (solid arrows), MVs (dashed arrows), and virions enclosed in MVs (arrowheads). Bars = 200 nm. (G) Immunoblots show the presence of integrin β-1, flotillin-1, LC3-II, CD81, and CD63 in MVs isolated from mock-infected (−) and infected (+) cells. The bands corresponding to the anti-HSV-1 antibody are also shown.

To determine whether the processes observed in HOG cells were similar in other cell types, we analyzed the MVs secreted by the human HeLa and MeWo cell lines, which are epithelial and melanoma cell lines, respectively. To ascertain whether MVs isolated from HeLa and MeWo cells infected with HSV-1 contained virions, MVs were isolated and processed for immunoelectron microscopy. In HeLa cells, MVs containing virions were observed in samples permeabilized with saponin and negatively stained and embedded with methylcellulose-uranyl acetate as described in Materials and Methods (Fig. 6C and D). Next, MVs from infected cells were layered onto HeLa cells at 4°C for 1 h and incubated for 15 min at 37°C before fixation and processing for electron microscopy as described in Materials and Methods. The presence of MVs and virions around the plasma membrane was detected (Fig. 6E), and occasionally, vesicles containing nucleocapsids were observed to be attached to the plasma membrane (Fig. 6F). Immunoblot assay showed the presence of integrin β-1, flotillin-1, CD63, CD81, and the autophagy marker LC3-II in MVs (Fig. 6G).

**FIG 7:**
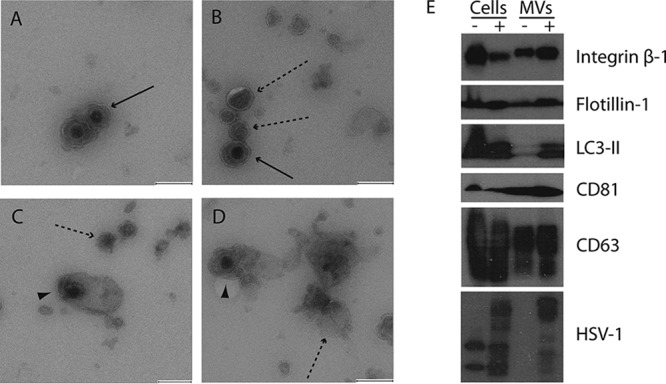
Analysis of MVs secreted by MeWo cells. MVs from MeWo cells infected with HSV-1 at an MOI of 1 for 24 h were isolated by differential centrifugation from the supernatants of MeWo cell cultures as previously described. (A to D) MVs were adsorbed onto collodion-carbon-coated copper grids and negatively stained and embedded with methylcellulose-uranyl acetate as described in Materials and Methods. Images show the presence of virions (solid arrows), MVs (dashed arrows), and virions enclosed in MVs (arrowheads). Bars = 200 nm. (E) Immunoblots show the presence of integrin β-1, flotillin-1, LC3-II, CD81, and CD63 in MVs isolated from mock-infected (−) and infected (+) cells. The bands corresponding to the anti-HSV-1 antibody are also shown. In this cell line, two nonspecific bands can be observed in the mock-infected cell lysate lane.

**FIG 8:**
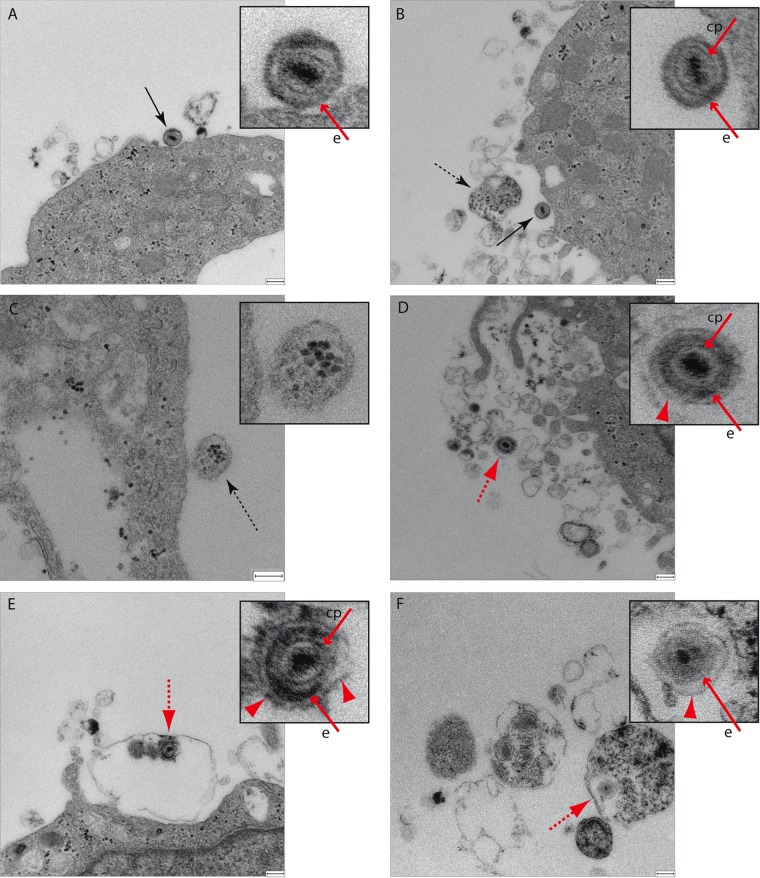
Electron microscopy of MeWo cells incubated with MVs. MVs from MeWo cells infected with HSV-1, isolated as described in the text, were layered onto MeWo cells and incubated at 4°C for 1 h. After that, they were incubated for 15 min at 37°C, fixed, and processed for electron microscopy. (A and B) Images show the presence of numerous MVs and virions (solid arrows). (B and C) MVs containing melanosomes (dashed arrows) were also found. (D to F) MVs containing virions (red dashed arrow) were occasionally observed. These virions contained a second envelope (red arrowhead), in addition to its viral envelope (e). Red solid arrows point to viral structural components: e, envelope; cp, capsid. Occasionally, in addition to virions, melanosomes were found in the same MV (F). Bars = 200 nm.

In MeWo cells, MVs containing virions were also observed (Fig. 7C and D), and like for HeLa and HOG cells, immunoblots showed the presence of integrin β-1, flotillin-1, CD63, CD81, and the autophagy marker LC3-II in MVs obtained from both infected and mock-infected cells (Fig. 7E). Nevertheless, unlike HeLa and HOG cells, MVs isolated from MeWo cells infected with HSV-1 contained virions enclosed in a second envelope (Fig. 8D to F), suggesting the presence of enveloped virions which exited the cell enclosed in shedding vesicles.

## DISCUSSION

MVs are EVs secreted by budding and shedding at the plasma membrane that participate in important biological processes and play significant roles in coagulation, inflammation, tumor progression, and other significant physiological and pathological processes (18, 58, 67–69). The involvement of these EVs in viral dissemination is being intensely studied, and currently, the significant role for MVs in the intercellular transmission of enveloped and nonenveloped viruses is widely accepted (48, 70–72). For instance, herpesviruses and retroviruses may recruit elements from the MV biogenesis pathways for functional virus release (47). However, MVs may either block or enhance infection or interfere with the immune system, which highlights the heterogeneity of MV function during viral infection (47). Using a coxsackievirus B3 (CVB3) strain modified to track infection and dissemination in real time, the release of MVs was recently studied after infection of neural progenitor and stem cells (NPSCs) and C2C12 myoblast cells. Infection of these cells induced the release of abundant MVs containing viral proteins and infectious virus, representing a novel route of virus dissemination (73).

Recent studies have shown the release of EVs with sizes ranging from 50 to 110 nm from HSV-1-infected cells (51, 74). These vesicles, which may have an effect on infection, carried viral and host transcripts—mRNAs, microRNAs, and noncoding RNAs—and proteins, which included innate immune components, such as the stimulator of interferon genes (STING) and the tetraspanins CD9, CD63, and CD81 (51, 74). The alleged functions of exosomes released from HSV-1-infected cells include priming the target cells and controlling the dissemination of the virus (51). However, to date, there is no evidence of the presence of HSV-1 virions within MVs.

Here we describe the features of MVs released by the human oligodendroglial HOG cell line infected with HSV-1 and report how these EVs could be involved in viral spread and immune evasion. To isolate MVs from the HOG cell supernatant, we used a differential centrifugation protocol (27, 75, 76). MVs are usually isolated by centrifugation at 10,000 to 20,000 × *g* (57–59), and the method used in the present study, centrifugation at 10,000 × *g* for 30 min, is a widely used protocol (56, 60–63). Electron microscopy observation of MVs derived from HSV-infected HOG cells revealed, for the first time, the presence of HSV-1 particles and structures in more than 5% of purified MVs. Immunostaining of these MVs with anti-HSV-1 antibodies coupled to colloidal gold particles confirmed the presence of HSV antigens in these MVs, whether or not these MVs were associated with identifiable virion structures.

One of the challenges of analyzing the role of EVs in virology is to separate the functional activity of vesicles from that of virions. Often, EVs isolated from infected cells may be very similar in size to viruses, and therefore, it may be very difficult to separate them (72). In general, EVs are highly heterogeneous in all parameters involved in differential centrifugation, and therefore, the complete separation of vesicles according to their diameter or density is still not possible (20, 27).

Our results indicate that the MV fraction isolated from infected HOG cells is not free of HSV-1 virions. This fact was considered when designing our experiments. First, to quantify MVs, we took advantage of the size difference between HSV-1 (<250 nm) and the larger and more heterogeneous MVs (up to 1,000 nm). To solve the similarity of size between virions and MVs (from 100 to 250 nm), we performed nanoparticle tracking analysis, establishing a threshold of 250 nm to analyze only the particles with a size larger than 250 nm, excluding, therefore, the presence of isolated virions in the MV fraction. For functional assays, we needed a more stringent method to discriminate infection mediated by MVs from infection mediated by free virions. A major strategy was to use CHO-K1 cells, which are nonpermissive for HSV-1 infection due to their lack of essential entry receptors (65, 77). Accordingly, our results showed that the infection of this cell line with HSV-1 yielded negligible viral productivity (Fig. 5D and E). In contrast, exposing CHO-K1 cells to MVs from infected HOG cells resulted in robust infection and viral production. Because CHO-K1 cells are not infected by free virions, we conclude that MVs can transfer infectivity to these cells. We also detected MVs in the cytosol of HOG cells (Fig. 4), suggesting that these vesicles can be phagocytosed or endocytosed. In gD receptor-negative CHO-K1 cells, virions get endocytosed but membrane fusion does not occur and capsids do not traffic to nuclei; instead, virions get degraded (77, 78). It is not known what endocytic process is used to internalize MVs, but capsid or genome delivery into the cytosol is independent of the presence of viral receptors for gD, such as nectin-1 or HVEM.

It has been argued that acquisition of an envelope might provide resistance to neutralizing antibodies, thus reinforcing viral spread (79), since neutralizing antibodies may be ineffective against virions protected within the MVs (73). Other studies have suggested that exosomes containing HCV might attach to target cells in the presence of virus-specific antibodies, making neutralizing antibodies against viral proteins ineffective in blocking infection (80). Therefore, virus-associated MVs might expand the natural tropism of viruses to target cells which lack canonical virus receptors, and in addition, neutralizing antibodies would be ineffective against virus sequestered within the protected environment of the MV, increasing its stability within the host during hematogenous spread (79). In this context and once it was demonstrated that CHO cells were susceptible to the MV-mediated infection with HSV-1, we wondered whether MVs would be detectable by antibodies since, as discussed above, neutralizing antibodies might be ineffective against virions protected within MVs (73, 79, 80). To evaluate whether the MVs isolated from HSV-1-infected HOG cells were susceptible to be detected by antibodies directed against HSV-1, we performed a standard neutralization assay. Our data showed a much less efficient neutralization for MVs than for virus (about 1 × 10E4 less efficient for MVs than for free virions); therefore, infection of CHO-K1 cells was not completely neutralized when virus-containing MVs were preincubated with potent neutralizing anti-HSV-1 antibodies. The lack of neutralization and the ability of MVs to infect nectin-1/HVEM-negative CHO-K1 cells suggest a novel way for HSV-1 to spread to and enter target cells.

The exact mechanistic process of HSV-1 targeting to microvesicles still remains unknown. Previous reports have shown the presence of autophagy markers, such as the lipidated form of LC3 (LC3-II), in virus-containing MVs, suggesting that the autophagic pathway contributes to the viral shedding (73). This finding coincides with the autophagosome-mediated exit without lysis (AWOL) model described for poliovirus release (81). Following this model, virus confined in double-membrane structures derived from the autophagic pathway could be transported into the extracellular milieu via fusion with the plasma membrane. The release of virus might occur either before or after fusion with endosomal or lysosomal organelles. We propose that a similar mechanism for the targeting of HSV-1 to MVs might be taking place in HOG cells. If this mechanism is occurring, it would be expected that MVs containing nonenveloped virions would be found. Interestingly, our experiments have shown that MVs, which are positive for the autophagy marker LC3-II, contain virions lacking an envelope, supporting our hypothesis. These results point, therefore, to a role for the autophagic pathway in MV-mediated HSV-1 dissemination, although future experiments will have to confirm that suggestion. Indeed, the role of autophagy in the HSV-1 cycle has been previously reported (82, 83). If this hypothesis were correct, the MVs would be produced via fusion of double-membrane vesicles with the plasma membrane instead of membrane shedding. In this situation, the MV fraction would comprise not only shedding MVs but also vesicles derived from the autophagic pathway. Therefore, the same methodology used to isolate vesicles might yield a heterogeneous population of vesicles deriving from different origins. In fact, the biochemical heterogeneity can be inferred from the results obtained by immunoblotting, which were positive not only for shedding MV markers, such as integrin β-1 and flotillin-1, but also for the exosomal markers CD81 and CD63 and the autophagy marker LC3-II.

Finally, to ascertain whether the processes observed in our oligodendrocytic model were similar in other cell types, we analyzed the MVs secreted by other human cell types, epithelial HeLa cells and the melanoma MeWo cell line. In both the HeLa and MeWo cell lines, MVs containing virions were observed, and like for HOG cells, immunoblots showed the presence of integrin β-1, flotillin-1, CD63, CD81, and the autophagy marker LC3-II in MVs derived from these cell lines. In HeLa cells, like in HOG cells, our findings point to a role for the autophagic pathway in MV-mediated HSV-1 dissemination, since MVs, which are positive for LC3-II, contain virions lacking an envelope. Nevertheless, unlike HeLa and HOG cells, MVs isolated from MeWo cells infected with HSV-1 contain virions enclosed in a second envelope, suggesting the presence of enveloped virions which have exited the cell enclosed in shedding vesicles. This indicates that the mechanistic process of HSV-1 targeting to MVs might be cell type dependent. Finally, a small number of MVs containing melanosomes, structures previously reported (84–86), were shown to enclose viral particles, indicating the existence of at least a common stage in their spread mechanism.

To summarize this study, we aimed at describing the features of MVs released by several cell lines infected with HSV-1 and their participation in the viral cycle. Our results indicate for the first time that MVs released by infected cells contain virions, are endocytosed by naive cells, and lead to a productive infection. Moreover, our results suggest that HSV-1 could spread through MVs to expand its tropism and possibly evade the host immune response. Finally, this study has been performed using human tumor cell lines. Future studies will have to be performed to confirm whether these results could be extrapolated to other, nontumor models.

## MATERIALS AND METHODS

### Antibodies and reagents.

Horseradish peroxidase-conjugated secondary anti-IgG antibodies were purchased from Millipore (Billerica, MA, USA). Alexa Fluor 647-conjugated secondary antibody was obtained from Molecular Probes (Eugene, OR, USA). Polyclonal rabbit anti-HSV-1 antibodies were from Dako and Antibodies Online GmbH. Mouse anti-PLP MAB388 and anti-integrin β-1 MAB5199 antibodies were from Millipore. Anti-CNPase C5922, low-glucose DMEM, fetal bovine serum (FBS), human insulin, triiodothyronine (T3), apo-transferrin, sodium selenite, putrescine, dibutyryl cyclic AMP (dbcAMP), and protease inhibitor cocktail were purchased from Sigma Chemical Co. (St. Louis, MO, USA). Anti-CD63 antibody 1B5 (87) was a kind gift from A. Fraile-Ramos (Universidad Complutense, Madrid, Spain). Anti-CD81 antibody 5A6 (SC-23962) was a kind gift from F. Sánchez-Madrid (Hospital de la Princesa, Madrid, Spain). The rabbit LC3-II antibody was from Novus Biologicals. Anti-flotillin-1 antibody 610821 was from BD Biosciences. Mowiol was from Calbiochem (Merck Chemicals, Germany).

### Cells and virus.

The HOG cell line, established from a surgically removed human oligodendroglioma (88), was kindly provided by A. T. Campagnoni (University of California, Los Angeles, USA). Cells were cultured on petri dishes in GM containing low-glucose DMEM supplemented with 10% fetal bovine serum (FBS), penicillin (50 U/ml), and streptomycin (50 μg/ml) at 37°C in an atmosphere of 5% CO_2_. To induce differentiation, cells were cultured in serum-free DM containing low-glucose DMEM supplemented with antibiotics and 50 μg/ml apo-transferrin, 0.5 mg/liter insulin, 30 nM triiodothyronine (T3), 30 nM sodium selenite, and 16.1 mg/liter putrescine. Cells cultured in this medium were also treated with 0.5 mM dbcAMP and 3-isobutyl-1-methylxanthine (IBMX) at a final concentration of 0.5 mM. The Chinese hamster ovary (CHO) cell line (subclone K1) was kindly provided by Richard Longnecker (Northwestern University).

Vero cells were kindly provided by Enrique Tabarés (Universidad Autónoma de Madrid). MeWo cells were a kind gift of L. Montoliu (CNB, Madrid, Spain). The HeLa human cell line (ATCC CCL2) was a kind gift of J. M. Almendral (Centre for Molecular Biology Severo Ochoa [CBMSO], Madrid, Spain). These cell lines were propagated in DMEM supplemented with 10% FBS, penicillin (50 U/ml), and streptomycin (50 μg/ml) at 37°C in an atmosphere of 5% CO_2_, as described previously (89). For isolation of MVs, MeWo and HeLa cells were cultured in DMEM supplemented with 1% exosome-depleted FBS and filtered through a 0.22-μm-pore-size Millex sterile syringe filters (Millipore).

HSV-1 K26-GFP was a kind gift of P. Desai (Johns Hopkins University, Baltimore, MD, USA). It was obtained by fusing GFP to the HSV-1 capsid protein VP26 (90). K26-GFP and wild-type HSV-1 (F strain; the GenBank accession number for the DNA genome sequence is GU734771) were propagated and titrated on Vero cells.

### Viral infections.

For viral infection assays, HOG cells were mock infected or infected with wild-type F or mutant K26-GFP virus diluted in serum-free low-glucose DMEM. During viral adsorption, cells were maintained in DMEM with antibiotics in the absence of fetal calf serum (FCS). Subsequently, cultures were rinsed and cultured in DM. The viral titer was quantified by an endpoint dilution assay determining the TCID_50_ in Vero cells, considering the final dilution that showed a cytopathic effect and using the Reed and Muench method.

### Immunoblot analysis.

Samples lysed in radioimmunoprecipitation assay buffer were mixed with 5× Laemmli sample buffer, subjected to SDS-PAGE in 10% acrylamide gels, and transferred to Immobilon-P membranes (Millipore). After blocking with 5% nonfat dry milk, 0.05% Tween 20 in phosphate-buffered saline (PBS), the blots were incubated for 1 h at room temperature with primary antibodies. After several washes with 0.05% Tween 20 in PBS, the blots were incubated for 1 h with secondary antibodies coupled to horseradish peroxidase, washed extensively, and developed using an enhanced chemiluminescence Western blotting kit (ECL; Amersham, Little Chalfont, UK).

### Immunofluorescence microscopy.

Cells grown on glass coverslips were fixed in 4% paraformaldehyde (PFA) for 20 min and rinsed with PBS. The cells were then permeabilized with 0.2% Triton X-100, rinsed, and incubated for 30 min with 3% bovine serum albumin in PBS. For double-labeled immunofluorescence analysis, cells were incubated for 1 h at room temperature with the appropriate primary antibody. Cells were then rinsed several times and incubated at room temperature for 30 min with the relevant fluorescent secondary antibody. Controls to assess labeling specificity included omission of the primary antibodies. After thorough washing, coverslips were mounted in Mowiol mounting medium. Images were obtained by the Optical and Confocal Microscopy Service of the Centre for Molecular Biology Severo Ochoa (CBMSO), using an LSM510 Meta system (Carl Zeiss) coupled to an inverted Axiovert 200 microscope. Processing of confocal images and colocalization analysis were done with Fiji-ImageJ software.

### Isolation of MVs.

HOG cells infected and mock infected with HSV-1 or K26-GFP were cultured with serum-free DM. At 24 h postinfection, 30 ml of supernatant was collected and transferred to polycarbonate bottles. MVs were isolated by differential centrifugation following a series of centrifugation steps at 4°C using a F0630 fixed-angle rotor (Beckman Coulter), first at 400 × *g* for 10 min, then at 2,500 × *g* for 15 min, and, finally, at 10,000 × *g* for 30 min. The pellet containing the MVs was washed with PBS, centrifuged once more at 10,000 × *g* for 30 min, and processed for the corresponding experiment.

### Staining MVs with PKH26.

MVs from noninfected HOG cells were stained with the red dye PKH26 following the manufacturer's (Sigma-Aldrich, St. Louis, MO) instructions. Briefly, MVs were isolated by differential centrifugation as previously described. After that, they were resuspended in 1 ml of diluent C (2× cell suspension). Then, we diluted 4 μl of PKH26 in 1 ml of diluent C (2× dye solution). After that we mixed the 2× cell suspension with the 2× dye solution and incubated the mixture for 5 min. The staining reaction was quenched by addition of 2 ml of FCS. Labeled cells were then centrifuged and resuspended in DMEM with 10% FCS for further analysis.

### Nanoparticle tracking analysis.

The pellet containing the MVs obtained as described above was resuspended and kept at 4°C in serum-free DM before its analysis by nanoparticle tracking analysis (NTA). For NTA we used a NanoSight LM10 system (NanoSight, Wiltshire, United Kingdom), which is equipped with fast video capture and particle-tracking software. Videos were collected and analyzed using the NTA software, version 2.3. For each measurement, the ambient temperature was recorded manually. One milliliter of each sample diluted 1:4 in Hanks balanced salt solution was injected into the NanoSight LM10 chamber. For each experimental condition, 2 samples were analyzed. For each sample, 4 videos of 90 s were recorded to obtain replicate histograms that were averaged. The histograms represent the concentration of particles (×10E8) per milliliter. To exclude the possibility of the presence of isolated virions, we performed the analysis of particles with a size larger than 250 nm.

### Electron microscopy.

To perform electron microscopy analysis of isolated MVs, the 10,000 × *g*-centrifugation-step pellets were resuspended in PBS containing 5 mmol/liter EDTA and fixed by adding an equal volume of 2% paraformaldehyde (PFA). Five to 10 μl of these fractions was adsorbed onto glow-discharged collodion-carbon-coated copper grids and negatively stained with 1% aqueous uranyl acetate for 45 s. Alternatively, MVs were stained and embedded with methylcellulose-uranyl acetate as described below.

Immunogold labeling of MVs onto the grids was performed at room temperature. MVs were permeabilized with 0.1% saponin for 5 min and incubated at room temperature with a rabbit polyclonal anti-HSV-1 antibody diluted 1,500 and 10 nm protein A-gold (Cell Microscopy Center, Utrecht University, Utrecht, The Netherlands). After immunolabeling, the samples were washed in distilled water and embedded in a mixture of 1.8% methylcellulose and 0.4% uranyl acetate at 4°C.

For conventional TEM, cells incubated with MVs were fixed with 4% PFA and 2% glutaraldehyde in 0.1 M phosphate buffer (PB; pH 7.4) for 90 min at room temperature. Postfixation was carried out with 1% OsO_4_ and 1% K_3_Fe(CN)_6_ in water at 4°C for 1 h. Samples were dehydrated with ethanol and *in situ* flat embedded in Taab 812 epoxy resin (Taab Laboratories) according to standard procedures. After polymerization, resin sheets containing the cell monolayers were detached from the substrate and mounted onto resin blocks to obtain orthogonal (from the bottom to the top of the cell) 80-nm ultrathin sections. The sections were deposited onto slot grids and stained with uranyl acetate and lead citrate.

Grids were examined at 80 kV in a JEOL JEM-1010 electron microscope, and all images were recorded with a TemCam-F416 (4K×4K) digital camera from Tietz Video and Image Processing Systems (TVIPS; Martinsried, Germany).

### Virus neutralization assay.

In the virus neutralization assay, we used a rabbit polyclonal anti-HSV-1 antibody from Antibodies Online GmbH (catalog number ABIN112743; Aachen, Germany). This antibody was produced with strain F of HSV-1 as an immunogen and presents cross-reactivity with HSV-2. It was purified by protein A chromatography, yielding a purity of >95%. To evaluate the neutralization capacity of the anti-HSV-1 antibody, an amount of 1 × 10E8 TCID_50_ was added to serial 1:2 dilutions of anti-HSV-1 antibody in 1.5-ml Eppendorf tubes. The volume was completed up to 200 μl with serum-free DMEM, and the mixtures were incubated for 1 h at room temperature with gentle agitation. A control without antibody was included. Then, the mixtures of antibody-virus were added to HOG cells cultured in a 24-well plate and adsorbed for 1 h at 37°C in a CO_2_ incubator. After adsorption, the cells were washed with serum-free DMEM and incubated in DM for 24 h in a 5% CO_2_ incubator. Infectivity was measured by the Reed and Muench method by the presence of a cytopathic effect on Vero cells. To perform the neutralization assays, we preincubated the virus or the MVs isolated from the supernatants of HSV-1 K26-GFP-infected HOG cells with the anti-HSV-1 antibody for 1 h at room temperature in a final volume of 200 μl of serum-free DMEM. After 1 h of preincubation, the mixture was layered on HOG or CHO cells in a 24-well plate and incubated at 37°C for 1 h. After adsorption, the inoculum was removed, the cells were washed with serum-free DMEM, and they were incubated at 37°C for 24 h in a 5% CO_2_ incubator.
